# Towards a Comprehensive Theory of Love: The Quadruple Theory

**DOI:** 10.3389/fpsyg.2020.00862

**Published:** 2020-05-19

**Authors:** Tobore Onojighofia Tobore

**Affiliations:** Independent Researcher, San Diego, CA, United States

**Keywords:** triangular theory of love, Romance, brand love, parental love, maternal love, Meaning of love, Definition of love, am I in love

## Abstract

Scholars across an array of disciplines including social psychologists have been trying to explain the meaning of love for over a century but its polysemous nature has made it difficult to fully understand. In this paper, a quadruple framework of attraction, resonance or connection, trust, and respect are proposed to explain the meaning of love. The framework is used to explain how love grows and dies and to describe brand love, romantic love, and parental love. The synergistic relationship between the factors and how their variations modulate the intensity or levels of love are discussed.

## Introduction

Scholars across an array of disciplines have tried to define the meaning and nature of love with some success but questions remain. Indeed, it has been described as a propensity to think, feel, and behave positively toward another (Hendrick and Hendrick, 1986). However, the application of this approach has been unsuccessful in all forms of love (Berscheid, 2010). Some social psychologists have tried to define love using psychometric techniques. Robert Sternberg Triangular Theory of Love and Clyde and Susan Hendrick’s Love Attitudes Scale (LAS) are notable attempts to employ the psychometric approach (Hendrick and Hendrick, 1986; Sternberg, 1986). However, data analysis from the administration of the LAS, Sternberg’s scale and the Passionate Love Scale by Hatfield and Sprecher’s (1986) found a poor association with all forms of love (Hendrick and Hendrick, 1989). Other studies have found a poor correlation between these and other love scales with different types of love (Whitley, 1993; Sternberg, 1997; Masuda, 2003; Graham and Christiansen, 2009).

In recent years, the neuropsychological approach to study the nature of love has gained prominence. Research has compared the brain activity of people who were deeply in love while viewing a picture of their partner and friends of the same age using functional magnetic resonance imaging (fMRI) and concluded that there is a specialized network of the brain involved in love (Bartels and Zeki, 2000). Indeed, several lines of investigation using fMRI have described a specialized area of the brain mediating maternal love (Noriuchi et al., 2008; Noriuchi and Kikuchi, 2013) and, fMRI studies have implicated multiple brain systems particularly the reward system in romantic love (Aron et al., 2005; Fisher et al., 2005, 2010; Beauregard et al., 2009). Brain regions including ventral tegmental area, anterior insula, ventral striatum, and supplementary motor area have been demonstrated to mediate social and material reward anticipation (Gu et al., 2019). Although brain imaging provides a unique insight into the nature of love, making sense of the psychological significance or inference of fMRI data is problematic (Cacioppo et al., 2003).

Also, there has been growing interests in the neurobiology of love. Indeed, evidence suggests possible roles for oxytocin, vasopressin, dopamine, serotonin, testosterone, cortisol, morphinergic system, and nerve growth factor in love and attachment (Esch and Stefano, 2005; De Boer et al., 2012; Seshadri, 2016; Feldman, 2017). However, in many cases, definite proof is still lacking and the few imaging studies on love are limited by selection bias on the duration of a love affair, gender and cultural differences (De Boer et al., 2012).

So, while advances have been made in unraveling the meaning of love, questions remain and a framework that can be employed to understand love in all its forms remains to be developed or proposed. The objective of this article is to propose a novel framework that can be applied to all forms of love.

## Theoretical Background and Hypothesis Development (The AAC Model)

In the past few decades, the psychological literature has defined and described different forms of love and from these descriptions, the role of attraction, attachment-commitment, and caregiving (AAC), appears to be consistent in all forms of love.

Attraction theory is one of the first approaches to explain the phenomenon of love and several studies and scholarly works have described the importance of attraction in different forms of love (Byrne and Griffitt, 1973; Berscheid and Hatfield, 1978; Fisher et al., 2006; Braxton-Davis, 2010; Grant-Jacob, 2016). Attraction has been described as an evolutionary adaptation of humans for mating, reproduction, and parenting (Fisher et al., 2002a, 2006).

The role of attachment in love has also been extensively investigated. Attachment bonds have been described as a critical feature of mammals including parent-infant, pair-bonds, conspecifics, and peers (Feldman, 2017). Indeed, neural networks including the interaction of oxytocin and dopamine in the striatum have been implicated in attachment bonds (Feldman, 2017). The key features of attachment include proximity maintenance, safety and security, and separation distress (Berscheid, 2010). Multiple lines of research have proposed that humans possess an innate behavioral system of attachment that is essential in love (Harlow, 1958; Bowlby, 1977, 1988, 1989; Ainsworth, 1985; Hazan and Shaver, 1987; Bretherton, 1992; Carter, 1998; Burkett and Young, 2012). Attachment is essential to commitment and satisfaction in a relationship (Péloquin et al., 2013) and commitment leads to greater intimacy (Sternberg, 1986).

Also, several lines of evidence have described the role of caregiving in love. It has been proposed that humans possess an inborn caregiving system that complements their attachment system (Bowlby, 1973; Ainsworth, 1985). Indeed, several studies have used caregiving scale and compassionate love scale, to describe the role of caring, concern, tenderness, supporting, helping, and understanding the other(s), in love and relationships (Kunce and Shaver, 1994; Sprecher and Fehr, 2005). Mutual communally responsive relationships in which partners attend to one another’s needs and welfare with the expectation that the other will return the favor when their own needs arise (Clark and Mills, 1979; Clark and Monin, 2006), have been described as key in all types of relationships including friendship, family, and romantic and compassionate love (Berscheid, 2010).

Attachment and caregiving reinforce each other in relationships. Evidence suggests that sustained caregiving is frequently accompanied by the growth of familiarity between the caregiver and the receiver (Bowlby, 1989, p. 115) strengthening attachment (Berscheid, 2010). Several studies have proposed that attachment has a positive influence on caregiving behavior in love and relationships (Carnelley et al., 1996; Collins and Feeney, 2000; Feeney and Collins, 2001; Mikulincer, 2006; Canterberry and Gillath, 2012; Péloquin et al., 2013).

The AAC model can be seen across the literature on love. Robert Sternberg triangular theory of love which proposes that love has three components —intimacy, passion, and commitment (Sternberg, 1986), essentially applies the AAC model. Passion, a key factor in his theory, is associated with attraction (Berscheid and Hatfield, 1978), and many passionate behaviors including increased energy, focused attention, intrusive thinking, obsessive following, possessive mate guarding, goal-oriented behaviors and motivation to win and keep a preferred mating partner (Fisher et al., 2002b, 2006; Fisher, 2005). Also, evidence indicates that attachment is central to intimacy, another pillar of the triangular theory (Morris, 1982; Feeney and Noller, 1990; Oleson, 1996; Grabill and Kent, 2000). Commitment, the last pillar of the triangular theory, is based on interdependence and social exchange theories (Stanley et al., 2010), which is connected to mutual caregiving and secure attachment.

Hendrick and Hendrick’s (1986), Love Attitudes Scale (LAS) which measures six types of love (Hendrick and Hendrick, 1986) is at its core based on the AAC model. Similarly, numerous works on love (Rubin, 1970; Hatfield and Sprecher, 1986; Fehr, 1994; Grote and Frieze, 1994), have applied one or all of the factors in the ACC model. Berscheid (2010), proposed four candidates for a temporal model of love including companionate love, romantic love, and compassionate love and adult attachment love. As described, these different types of love (romantic, companionate, compassionate, and attachment) all apply at least one or all of the factors in the AAC model.

## New Theory (The Quadruple Framework)

The AAC model can be fully captured by four fundamental factors; attraction, connection or resonance, trust, and respect, providing a novel framework that could explain love in all its forms. Table 1 shows the core factors of love, and the four factors derived from them.

**TABLE 1:** Factors of love.

Core factors	Factors of love	Strengthening or driving factors	Behavioral traits
Attraction Attachment	Attraction	Physical attributes, personality, wealth, value, etc.	Passion, intimacy, commitment.
Attachment-Commitment Caregiving	Connection/resonance	Similarity, proximity, familiarity, positive shared experiences, interdependence, novelty.	Friendship, separation distress, worry, and concern, commitment and Intimacy, compassion or caregiving.
Attachment-Commitment Caregiving	Trust	Reliability, familiarity, mutual self-disclosures, positive shared experiences.	Intimacy, commitment, compassion or caregiving
Attachment-Commitment Caregiving	Respect	Reciprocal appreciation, admiration, consideration, concern for wellbeing, and tolerance	Commitment, intimacy, compassion or caregiving

### Attraction

Evidence suggests that both attachment and attraction play a role in obsession or passion observed in love (Fisher et al., 2005; Honari and Saremi, 2015). Attraction is influenced by the value or appeal perceived from a relationship and this affects commitment (Rusbult, 1980).

### Connection or Resonance

Connection is key to commitment, caregiving, and intimacy. It creates a sense of oneness in relationships and it is strengthened by proximity, familiarity, similarity, and positive shared experiences (Sullivan et al., 2011; Beckes et al., 2013). Homogeneity or similarity has been observed to increase social capital and engagement among people (Costa and Kahn, 2003a, b), and it has been described as foundational to human relationships (Tobore, 2018, pp. 6–13). Research indicates that similarity plays a key role in attachment and companionship as people are more likely to form long-lasting and successful relationships with those who are more similar to themselves (Burgess and Wallin, 1954; Byrne, 1971; Berscheid and Reis, 1998; Lutz-Zois et al., 2006). Proximity plays a key role in caregiving as people are more likely to show compassion to those they are familiar with or those closest to them (Sprecher and Fehr, 2005). Similarity and proximity contribute to feelings of familiarity (Berscheid, 2010). Also, caregiving and empathy are positively related to emotional interdependence (Hatfield et al., 1994).

### Trust

Trust is crucial for love (Esch and Stefano, 2005) and it plays an important role in relationship intimacy and caregiving (Rempel and Holmes, 1985; Wilson et al., 1998; Salazar, 2015), as well as attachment (Rodriguez et al., 2015; Bidmon, 2017). Familiarity is a *sine qua non* for trust (Luhmann, 1979), and trust is key to relationship satisfaction (Simpson, 2007; Fitzpatrick and Lafontaine, 2017).

### Respect

Respect is cross-cultural and universal (Frei and Shaver, 2002; Hendrick et al., 2010) and has been described as fundamental in love (Hendrick et al., 2011). It plays a cardinal role in interpersonal relations at all levels (Hendrick et al., 2010). Indeed, it is essential in relationship commitment and satisfaction (Hendrick and Hendrick, 2006) and relationship intimacy and attachment (Alper, 2004; Hendrick et al., 2011).

## Synergetic Interactions of the Four Factors

### Connection and Attraction

Similarity, proximity, and familiarity are all important in connection because they promote attachment and a sense of oneness in a relationship (Sullivan et al., 2011; Beckes et al., 2013). Research indicates that proximity (Batool and Malik, 2010) and familiarity positively influence attraction (Norton et al., 2015) and several lines of evidence suggests that people are attracted to those similar to themselves (Sykes et al., 1976; Wetzel and Insko, 1982; Montoya et al., 2008; Batool and Malik, 2010; Collisson and Howell, 2014). Also, attraction mediates similarity and familiarity (Moreland and Zajonc, 1982; Elbedweihy et al., 2016).

### Respect and Trust

Evidence suggests that respect promotes trust (Ali et al., 2012).

### Connection, Respect, Trust, and Attraction

Trust affects attraction (Singh et al., 2015). Trust and respect can mediate attitude similarity and promote attraction (Singh et al., 2016).

So, although these factors can operate independently, evidence suggests that the weakening of one factor could negatively affect the others and the status of love. Similarly, the strengthening of one factor positively modulates the others and the status of love.

## Love Cycle

**FIGURE 1:**
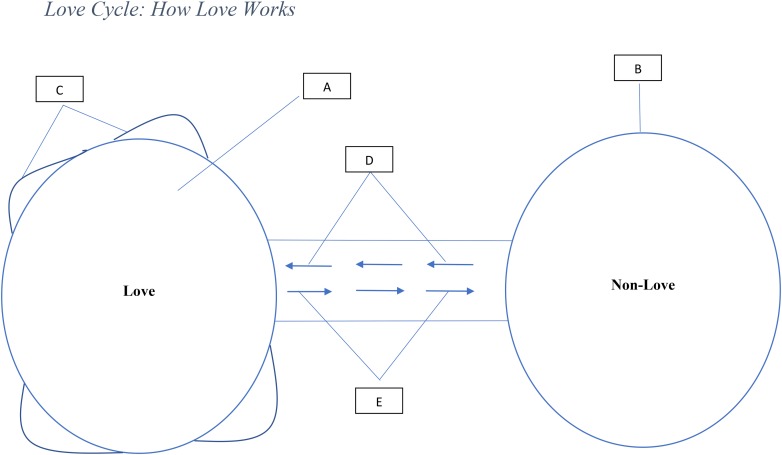
Description: **(A)** Presence of love (all factors are present). **(B)** Absence of love (state of non-love or state where all factors are latent or dormant). **(C)** Different levels of love due to variations in the four factors. **(D)** Movement from non-love toward love (developmental stage: at least one but not all four factors are present). **(E)** Movement away from love toward non-love (decline stage: at least one or more of the four factors are absent).

Relationships are dynamic and change as events and conditions in the environment change (Berscheid, 2010). Love is associated with causal conditions that respond to these changes favorably or negatively (Berscheid, 2010). In other words, as conditions change, and these factors become present, love is achieved and if they die, it fades. Figure 1 below explains how love grows and dies. Point C in the figure explains the variations in the intensity or levels of love and this variation is influenced by the strength of each factor. The stronger the presence of all factors, the higher the intensity and the lower, the weaker the intensity of love. The concept of non-love is similar to the “non-love” described in Sternberg’s triangular theory of love in which all components of love are absent (Sternberg, 1986).

## Application of the Quadruple Framework on Romantic, Brand and Parental Love

Romantic, parental and brand love have been chosen to demonstrate the role of these factors and their interactions in love because there is significant existing literature on them. However, they can be applied to understand love in all its forms.

### Romantic Love

#### Attraction and Romantic Love

Attraction involves both physical and personality traits (Braxton-Davis, 2010; Karandashev and Fata, 2014). To this end, attraction could be subdivided into sexual or material and non-sexual or non-material attraction. Sexual or material attraction includes physical attributes such as beauty, aesthetics, appeal, wealth, etc. In contrast, non-sexual or non-material attraction includes characteristics such as personality, social status, power, humor, intelligence, character, confidence, temperament, honesty, good quality, kindness, integrity, etc. Both types of attraction are not mutually exclusive.

Romantic love has been described as a advanced form of human attraction system (Fisher et al., 2005) and it fits with the passion component of Sternberg’s triangular theory of love which he described as the quickest to recruit (Sternberg, 1986). Indeed, research indicates that physical attractiveness and sensual feelings are essential in romantic love and dating (Brislin and Lewis, 1968; Regan and Berscheid, 1999; Luo and Zhang, 2009; Braxton-Davis, 2010; Ha et al., 2010; Guéguen and Lamy, 2012) and sexual attraction often provides the motivational spark that kickstarts a romantic relationship (Gillath et al., 2008). Behavioral data suggest that love and sex drive follow complementary pathways in the brain (Seshadri, 2016). Indeed, the neuroendocrine system for sexual attraction and attachment appears to work synergistically motivating individuals to both prefer a specific mating partner and to form an attachment to that partner (Seshadri, 2016). Sex promotes the activity of hormones involved in love including arginine vasopressin in the ventral pallidum, oxytocin in the nucleus accumbens and stimulates dopamine release which consequently motivates preference for a partner and strengthens attachment or pair-bonding (Seshadri, 2016).

Also, romantic love is associated with non-material attraction. Research indicates that many people are attracted to their romantic partner because of personality traits like generosity, kindness, warmth, humor, helpfulness, openness to new ideas (Giles, 2015, pp. 168–169). Findings from a research study on preferences in human mate selection indicate that personality traits such as kindness/considerate and understanding, exciting, and intelligent are strongly preferred in a potential mate (Buss and Barnes, 1986). Indeed, character and physical attractiveness have been found to contribute jointly and significantly to romantic attraction (McKelvie and Matthews, 1976).

Attraction is key to commitment in a romantic relationship (Rusbult, 1980), indicating that without attraction a romantic relationship could lose its luster. Also, romantic attraction is weakened or declines as the reason for its presence declines or deteriorates. If attraction is sexual or due to material characteristics, then aging or any accident that compromises physical beauty would result in its decline (Braxton-Davis, 2010). Loss of fortune or social status could also weaken attraction and increase tension in a relationship. Indeed, tensions about money increase marital conflicts (Papp et al., 2009; Dew and Dakin, 2011) and predicted subsequent divorce (Amato and Rogers, 1997).

#### Connection and Romantic Love

Connection or resonance fits with the intimacy, and commitment components of Sternberg’s triangular theory of love (Sternberg, 1986). Connection in romantic love involves intimacy, friendship or companionship and caregiving and it is strengthened by novelty, proximity, communication, positive shared experiences, familiarity, and similarity. It is what creates a sense of oneness between romantic partners and it is expressed in the form of proximity seeking and maintenance, concern, and compassion (Neto, 2012). Evidence suggests that deeper levels of emotional involvement or attachment increase commitment and cognitive interdependence or tendency to think about the relationship in a pluralistic manner, as reflected in the use of plural pronouns to describe oneself, romantic partner and relationship (Agnew et al., 1998).

Research indicates that both sexual attraction and friendship are necessary for romantic love (Meyers and Berscheid, 1997; Gillath et al., 2008; Berscheid, 2010), indicating that connection which is essential for companionship plays a key role in romantic love. A study on college students by Hendrick and Hendrick (1993) found that a significant number of the students described their romantic partner as their closest friend (Hendrick and Hendrick, 1993), reinforcing the importance of friendship or companionship in romantic love.

Similarity along the lines of values, goals, religion, nationality, career, culture, socioeconomic status, ethnicity, language, etc. is essential in liking and friendship in romantic love (Berscheid and Reis, 1998). Research indicates that a partner who shared similar values and interests were more likely to experience stronger love (Jin et al., 2017). Indeed, the more satisfied individuals were with their friendships the more similar they perceived their friends to be to themselves (Morry, 2005). Also, similarity influences perceptions of familiarity (Moreland and Zajonc, 1982), and familiarity plays a role in the formation of attachment and connectedness because it signals safety and security (Bowlby, 1977). Moreover, similarity and familiarity affect caregiving. Sprecher and Fehr (2005), found compassion or caregiving were lower for strangers, and greatest for dating and marital relationships, indicating that similarity and familiarity enhance intimacy and positively influences caregiving (Sprecher and Fehr, 2005).

Proximity through increased exposure is known to promote liking (Saegert et al., 1973), familiarity and emotional connectedness (Sternberg, 1986; Berscheid, 2010). Exposure through fun times and direct and frequent communication is essential to maintaining and strengthening attachment and connectedness (Sternberg and Grajek, 1984). In Sternberg’s triangular theory, effective communication is described as essential and affects the intimacy component of a relationship (Sternberg, 1986). Indeed, intimacy grows from a combination of mutual self-disclosure and interactions mediated by positive partner responsiveness (Laurenceau et al., 1998, 2005; Manne et al., 2004), indicating that positive feedback and fun times together strengthens connection.

Also, sexual activity is an important component of the reward system that reinforces emotional attachment (Seshadri, 2016), indicating that sexual activity may increase emotional connectedness and intimacy. Over time in most relationships, predictability grows, and sexual satisfaction becomes readily available. This weakens the erotic and emotional experience associated with romantic love (Berscheid, 2010). Research shows that a reduction in novelty due to the monotony of being with the same person for a long period is the reason for this decline in sexual attraction (Freud and Rieff, 1997, p. 57; Sprecher et al., 2006, p. 467). According to Sternberg (1986), the worst enemy of the intimacy component of love is stagnation. He explained that too much predictability can erode the level of intimacy in a close relationship (Sternberg, 1986). So, novelty is essential to maintaining sexual attraction and strengthening connection in romantic love.

Jealousy and separation distress which are key features of romantic love (Fisher et al., 2002b), are actions to maintain and protect the emotional union and are expressions of a strong connection. Research has found a significant correlation between anxiety and love (Hatfield et al., 1989) and a positive link between romantic love and jealousy in stable relationships (Mathes and Severa, 1981; Aune and Comstock, 1991; Attridge, 2013; Gomillion et al., 2014). Indeed, individuals who feel strong romantic love tend to be more jealous or sensitive to threats to their relationship (Orosz et al., 2015).

Connection in romantic love is weakened by distance, a dearth of communication, unsatisfactory sexual activity, divergences or dissimilarity of values and interests, monotony and too much predictability.

#### Trust and Romantic Love

Trust is the belief that a partner is, and will remain, reliable or dependable (Cook, 2003). Trust in romantic love fits with the intimacy, and commitment components of Sternberg’s triangular theory of love which includes being able to count on the loved one in times of need, mutual understanding with the loved one, sharing of one’s self and one’s possessions with the loved one and maintaining the relationship (Sternberg, 1986).

It has been proposed that love activates specific regions in the reward system which results in a reduction in emotional judgment and fear (Seshadri, 2016). This reduced fear or trust has been identified as one of the most important characteristics of a romantic relationship and essential to fidelity, commitment, monogamy, emotional vulnerability, and intimacy (Laborde et al., 2014). Indeed, trust can deepen intimacy, increase commitment and increase mutual monogamy, and make a person lower their guards in the belief that they are safe from harm (Larzelere and Huston, 1980; Bauman and Berman, 2005). People with high trust in romantic relationships tend to expect that their partner will act in their interest causing them to prioritize relationship dependence over making themselves invulnerable from harm or self-protection (Luchies et al., 2013). In contrast, people with low trust in their partner tend to be unsure about whether their partner will act in their interests and prioritize insulating themselves from harm over relationship dependence (Luchies et al., 2013).

Trust takes time to grow into a romantic relationship. Indeed, people in a relationship come to trust their partners when they see that their partner’s action and behavior moves the relationship forward or acts in the interest of the relationship and not themself (Wieselquist et al., 1999). Research indicates that trust is associated with mutual self-disclosure (Larzelere and Huston, 1980), and positive partner responsiveness which are both essential to the experience of friendship and intimacy in romantic relationships (Larzelere and Huston, 1980; Reis and Shaver, 1988; Laurenceau et al., 1998).

Also, trust influences caregiving and compassion. Evidence suggests that compassion is positively related to trust (Salazar, 2015). Mutual communal responsiveness or caregiving in relationships in which partners attend to one another’s needs and welfare is done because they are confident that the other will do the same when or if their own needs arise (Clark and Monin, 2006). Repeated acts of communal responsiveness given with no expectation of payback provide a partner with a sense of security and trust and increase the likelihood that they will be communally responsive if or when the need arises (Clark and Monin, 2006), and contributes to a sense of love in romantic relationships (Berscheid, 2010).

Loss or weakening of trust could spell the end of romantic love. Indeed, mistrust corrupts intimacy and often indicates that a relationship has ended or near its end (LaFollette and Graham, 1986) and it makes mutual monogamy, and commitment difficult to achieve in a romantic relationship (Towner et al., 2015). A study on individuals who had fallen out of romantic love with their spouse found that loss of trust and intimacy was part of the reason for the dissolution of love (Sailor, 2013).

#### Respect and Romantic Love

Multiple lines of evidence suggest that respect is expected in both friendships and romantic relationships (Gaines, 1994, 1996). In romantic love, it entails consideration, admiration, high regard, and value for the loved one as a part of one’s life (Sternberg and Grajek, 1984; Hendrick et al., 2011).

Gottman (1999), found that the basis for a stable and satisfactory marital relationship is friendship filled with fondness and admiration (Gottman, 1999). Respect is considered one of the most important things married couples want from their partner (Gottman, 1994). Grote and Frieze (1994), found that respect correlates with companionate or friendship love (Grote and Frieze, 1994), indicating that respect is essential to intimacy and relationship satisfaction. Also, respect is positively correlated with passion, altruism, self-disclosure, and relationship overall satisfaction (Frei and Shaver, 2002; Hendrick and Hendrick, 2006). It is associated with the tendency to overlook a partner’s negative behavior or respond with pro-relationship actions or compassion to their shortcomings (Rusbult et al., 1998; Gottman, 1999).

Absence or a lack of respect could spell the end of romantic love. Research indicates that there is an expectation of mutual respect in friendship and most relationships and people reacted negatively when this expectation is violated (Hendrick et al., 2011), indicating that a lack of respect could negatively affect commitment and attraction. Indeed, denial of respect is an important negative behavior in friendships and most relationships (Gaines, 1994, 1996) and a lack of respect is a violation of what it means to love one ‘s partner in a close romantic relationship (Hendrick et al., 2011). Gottman (1993, 1994) identified contempt, criticism, defensiveness, and stonewalling as four of the relationally destructive behavior and he labeled them as “the four horsemen of the apocalypse.”

##### Romantic love summary

Romantic love involves the interactions and synergistic interplay between respect, connection, trust, and attraction. All four must be present in love. Any event that results in the loss of any of these factors could cause romantic love to gradually decline and unless effort is made to replenish it, it will eventually fade or collapse. Romantic love is dynamic and requires significant investment from both partners to keep it alive.

### Parental Love

#### Attraction and Parental Love

Attraction plays an essential role in parental love and it could be material or non-material. Material attraction involves the child’s health, gender, accomplishments or success, and attractiveness. In contrast, non-material attraction includes traits such as intelligence, character, and other personality traits.

Evidence suggests that culture influences gender preference with attraction greater for sons in most cases (Cronk, 1993). Indeed, mothers and fathers have been found to favor the more intelligent and more ambitious/industrious child (Lauricella, 2009). Also, parental perception that investment in a child will cost more than the benefits to be gained from taking care of the child might influence negative behavior toward the child. Indeed, multiple lines of evidence suggest that parental unemployment increases the rates of child maltreatment and abuse (Steinberg et al., 1981; Lindo et al., 2013). Research indicates that teen mothers who have poor social support reported greater unhappiness, were at greater risk for child abuse and often employed the use of physical punishment toward their child (Haskett et al., 1994; de Paúl and Domenech, 2000).

Also, several studies have suggested that parents tended to favor healthy children (Mann, 1992; Barratt et al., 1996; Hagen, 1999). However, when resources are plentiful, parents tend to invest equally in less healthy or high-risk children (Beaulieu and Bugental, 2008), because they have abundant resources to go around without compromising the reproductive value of healthy children (Lauricella, 2009).

#### Connection and Parental Love

Connection creates a sense of oneness between parent and child and involves caregiving, intimacy, and attachment. It is influenced by proximity, positive and unique shared experiences, and similarity along virtually every dimension between parent and child.

Proximity, and similarity increases attachment and intimacy between parent and child. Research shows that parents are perceived as favoring genetically related children (Salmon et al., 2012), and evidence suggests that paternal resemblance predicted paternal favoritism (Lauricella, 2009). Parental proximity and similarity to a biological child are unique because it is based on genes and blood. In contrast, intimacy between a parent and an adopted child is based solely on shared experiences and proximity and takes time to grow and on many occasions may not develop (Hooks, 1990; Hughes, 1999).

Dissimilarities or discrepancy in values, attitudes, etc., can create problems between children and parents and can have a profound effect on their relationship. Indeed, evidence suggests that the rebel child tended to be less close to the parents (Rohde et al., 2003). Research has found that adolescents who are less religious than their parents tend to experience lower-quality relationships with their parents which results in higher rates of both internalizing and externalizing symptoms (Kim-Spoon et al., 2012). When parents and family members were very religious, and a child comes out as an atheist, relationship quality could suffer in the form of rejection, anger, despair, or an inability to relate to one another (Zimmerman et al., 2015). A study of lesbian, gay, and bisexual youngsters, for patterns of disclosure of sexual orientation to families, found that those who had disclosed reported verbal and physical abuse by parents and family members (D’Augelli et al., 1998). Honor killing of female children which have been reported in Pakistan and some parts of the Middle East because of deviation from traditional gender roles or crossing of social boundaries that are deemed as taboo in their culture (Lindsey and Sarah, 2010), is another example of the negative effects of the discrepancy in values between parents and child.

Unique shared experiences between parent and child could increase connection. Bank (1988) observed that the development of favoritism seems to require that the “child’s conception or birth be unusual or stressful,” (Bank, 1988). Evidence suggests that parents most favored child tended to be last-born child and this is linked to their unique position, vulnerability and neediness (Rohde et al., 2003). Also, proximity, positive experiences and time spent together increases connection and intimacy. Research indicates that parents tend to give more love and support to the grown child they were historically closest to and got along with (Siennick, 2013). A study of primiparous women found that mothers with greater contact with their infants were more reluctant to leave them with someone else, and engaged more intimately with their child (Klaus et al., 1972).

Divorce could create distance between a parent and child, weakening connection and intimacy. Indeed, one of the outcomes of divorce is the lessening of contact between divorced non-custodial fathers and their children (Appleby and Palkovitz, 2007), and this can reduce intimacy (Guttmann and Rosenberg, 2003).

Also, parental separation distress, worry, and concern for their child’s welfare, academic performance, and future are expressions of connection and a lack thereof is a sign of poor connection. Indeed, the levels of concern and worry expressed between children and their parents influenced their perceptions of the relationship quality (Hay et al., 2007).

#### Trust and Parental Love

Trust is essential to parental attachment, intimacy, and caregiving. When there is mistrust, attachment and intimacy between a parent and their child are disrupted or unable to blossom. In Africa and many parts of the world, there have been reports of children being condemned and abandoned by their parents simply because they are tagged as witches with mysterious evil powers (Tedam, 2014; Bartholomew, 2015; Briggs and Whittaker, 2018). The tag of “witchcraft” stirs up fear and anger, causing the child to be perceived as a deadly threat which inevitably damages attachment, intimacy and eliminates the need for caregiving.

Research has found that firstborn children were most likely to be chosen as those to whom mothers would turn when facing personal problems or crises (Suitor and Pillemer, 2007). This tendency may be linked to trust. Moreover, evidence suggests that the rebel child tended to be less close to the parents (Rohde et al., 2003). In other words, the more obedient, and reliable child is likely to gain the confidence and intimacy of the parents. In contrast, the disobedient and unreliable child is excluded or kept at a distance. Also, trust and poor connection could influence inheritance and disinheritance decisions. Indeed, estrangement, alienation and disaffection of a parent toward a child could result in disinheritance (Batts, 1990; Brashier, 1994, 1996; Foster, 2001; Arroyo et al., 2016).

#### Respect and Parental Love

Respect in parental love entails treating the child with consideration and regard. This consideration and regard for the child are essential to intimacy, caregiving and attachment. Indeed, respect is foundational to a harmonious relationship between parent and child (Dixon et al., 2008). Evidence suggests that humans possess an innate behavioral system that leads them to form an attachment to a familiar person who provides care, comfort, and protection (Harlow, 1958; Bowlby, 1989). Repeated acts of caregiving contribute to a sense of love in all types of relationships (Berscheid, 2010), reinforcing the role of parental caregiving in fostering intimacy and attachment with the child.

Taking care of an infant’s needs, and making sure they are safe and well, all fall under consideration and regard for the child. Child abuse and neglect (Tedam, 2014; Bartholomew, 2015; Briggs and Whittaker, 2018), is a display of a lack of consideration for the child’s need.

Also, respect in parental love involves admiration. Research has found that fathers treated more ambitious/industrious sons with high regard, and both parents favored the more intelligent and more ambitious/industrious daughters (Lauricella, 2009) indicating that a child that engages in activities or behavior that is highly regarded by their parents may gain favor with their parents, strengthening intimacy and vice versa.

##### Parental love summary

Parental love involves the interactions and synergistic interplay between respect, connection, trust, and attraction. Any event that results in the loss of any of these factors could cause parental love to gradually decline. In many cases, the behavior and actions of a child significantly influence parental love.

### Brand Love

Brand love has been defined as the level of passionate emotional attachment a satisfied or happy consumer has for a brand and evidence suggests it is very similar to interpersonal love (Russo et al., 2011).

#### Attraction and Brand Love

Attraction plays an essential role in brand love. Material attraction for a brand includes attributes like superior design, quality, and aesthetics, price, benefits, etc. Non-material attraction involves social status symbol, brand personality, uniqueness, distinctiveness, user experience, image, etc. evidence suggests that when talking about loved brands, people often talk passionately about the brand’s many attractive qualities such as its exceptional performance, good-looking design, value for money, and other positive attributes (Fournier, 1998; Whang et al., 2004; Carroll and Ahuvia, 2006; Batra et al., 2012). Research on brand love has found that brand attractive attributes such as prestige or uniqueness influence brand passion which affects relevant factors such as purchase intention (Bauer et al., 2007).

Also, brand attraction influences brand loyalty, and commitment. Indeed, research indicates that brand benefits influences brand loyalty or commitment (Huang et al., 2016). Brand personality (image, distinctiveness, and self-expressive value) is strongly associated with brand identification and loyalty (Kim et al., 2001; Elbedweihy et al., 2016).

#### Connection and Brand Love

Connection is essential to brand love. It involves brand attachment, commitment, and intimacy and it is strengthened by brand identification, image, familiarity or awareness, proximity, length or frequency of usage and similarity or congruences along virtually every dimension including values, lifestyle, goals, etc. between brand and customer. Brand awareness which means brand familiarity has been described as essential for people to identify with a brand (Pascual and Académico, 2015), and it indirectly affects current purchases (Esch et al., 2006).

Also, brand identification promotes a sense of oneness between a brand and a customer strengthening commitment and it is driven by brand self-similarity, brand prestige and brand distinctiveness (Stokburger-Sauer et al., 2008). Indeed, brand identification contributes to the development of brand love and brand loyalty (Alnawas and Altarifi, 2016) and brand image and identification influence loyalty and positive word of mouth (Carroll and Ahuvia, 2006; Batra et al., 2012; Anggraeni and Rachmanita, 2015). Brand identity, values and lifestyle similarities to those of the customer appear to have a strong and significant relationship with brand love (Batra et al., 2012; Rauschnabel and Ahuvia, 2014; Alnawas and Altarifi, 2016; Elbedweihy et al., 2016). Findings from research suggest that customer-to-customer similarity and sense of community drive consumer brand identification, loyalty, and engagement (Bergkvist and Bech-Larsen, 2010; Elbedweihy et al., 2016).

Moreover, proximity and interaction play a role in brand love. Indeed, the duration of the relationship between a customer and a brand is essential in brand love (Albert et al., 2007). Fournier (1998), discussed interdependence which involved frequent brand interactions as necessary for a strong brand relationship (Fournier, 1998). Similarly, Batra et al. (2012) found that having a long-term relationship, positive emotional connection and frequent interactions with a brand was an important aspect of brand love (Batra et al., 2012). Indeed, shared experiences and history between a person and a brand can increase their emotional attachment, make the brand to become an important part of the person’s identity narrative and increases their loyalty to the brand (Thomson et al., 2005; Pedeliento et al., 2016).

Just like romantic love, concern and worry and proximity seeking, or maintenance are an expression of emotional connectedness to the brand. Indeed, anticipated separation distress has been described as a core element of brand love (Batra et al., 2012), and consumers are likely to feel strong desires to maintain proximity with their loved objects, even feeling “separation distress” when they are distanced from them (Thomson et al., 2005; Park et al., 2010).

Also, novelty through continued innovation is vital to maintaining and strengthening both attraction and connection. According to the Harvard business review, the relationship between brand and consumer go through “ruts” and to “keep the spark” alive, innovation and news are essential (Halloran, 2014). Research indicates that innovation plays a role in brand equity and it impacts brand identification or resonance (Sinha, 2017).

Lack of brand familiarity or awareness, poor or negative user experience, a dearth of innovation and increased dissimilarities in values and lifestyles between brand and consumer can all weaken brand connection.

#### Trust and Brand Love

Trust is essential to brand attachment, intimacy, and commitment. It involves confidence and reliability, or dependability of the brand and it is influenced by brand image, familiarity, values, user experience, and quality. Indeed, brand trust directly influences brand love (Turgut and Gultekin, 2015; Meisenzahl, 2017) and a strong relationship exists between brand love and brand trust and identification (Albert and Merunka, 2013). Evidence suggests that brand familiarity influences brand trust (Ha and Perks, 2005) and brand trust and experience, positively influence brand attachment (Erciş et al., 2012; Chinomona, 2013; Chinomona and Maziriri, 2017).

Also, brand trust affects brand purchase, loyalty, and commitment. Evidence suggests that a strong relationship exists between brand love and brand trust, brand commitment, positive word of mouth, and willingness to pay a higher price for the brand (Albert and Merunka, 2013). Research indicates that brand trust positively affects brand loyalty (Setyawan and Kussudiyarsana, 2015), directly influences brand purchase intentions (Yasin and Shamim, 2013) and positively influences current and future purchases (Erciş et al., 2012). Indeed, more than any other factor, brand trust has been identified as essential for future purchases of a brand (Esch et al., 2006). It is essential in determining purchase loyalty and attitudinal loyalty and it plays a role in brand market share (Chaudhuri and Holbrook, 2001). Brand trust affects both affective and continuance commitment and affective commitment influences repurchase intention and loyalty (Erciş et al., 2012).

Brand quality is essential to brand trust and love. Indeed, Fournier (1998), discussed the role of brand quality in brand love and highlighted the role of trust in relationship satisfaction and strength (Fournier, 1998). Also, brand trust has been found to positively affect resistance to negative information and repurchase intention (Turgut and Gultekin, 2015).

Brand trust is weakened by poor user experience, brand quality, brand image, and a lack of brand familiarity.

#### Respect and Brand Love

Brand respect is essential in brand love and plays an important role in brand attachment, intimacy, and commitment. It is influenced by brand identification, values, image, experience, and quality. Brand respect is displayed by the customer in the form of high regard, admiration for the brand, brand loyalty and consideration or tolerance of negative information. Indeed, brand familiarity positively affects brand respect (Zhou, 2017), indicating that brand familiarity increases regard for a brand. Evidence suggests that brand image positively influences brand respect and love (Cho, 2011), indicating that brand image modulates a customer’s regard and admiration for a brand.

Brand respect influences brand commitment and loyalty. Indeed, a strong relationship has been found between brand respect and brand loyalty (Cho, 2011) and brand admiration results in greater brand loyalty, stronger brand advocacy, and higher brand equity (Park et al., 2016). Brand respect affects the behavioral outcomes of brand love such as affective commitment, and willingness to pay a price premium (Garg et al., 2016; Park et al., 2016).

Also, evidence suggests that customers’ admiration or high regard for a brand contributes to why they tend to ignore negative information about the brand (Elbedweihy et al., 2016). Fournier (1998), included respect as one of the components of brand partner quality. This means that respect is one of the factors that reflects the consumer’s evaluation of the brand’s performance (Fournier, 1998).

A lack of respect could negatively influence the relationship between a brand and a customer. Indeed, people react negatively when the expectation of respect is violated (Hendrick et al., 2011) and a violation of expectation between brand and customer has been found to contribute to brand hate (Zarantonello et al., 2016).

##### Brand love summary

Brand love involves the interactions and synergistic interplay between respect, connection, trust, and attraction. Any event that results in the loss of any of these factors could cause brand love to gradually decline and unless effort is made to replenish it, it will eventually fade or collapse. Brand love is dynamic and requires significant investment from the brand to keep it alive.

## Strengths and Advances Made by the Quadruple Theory

The quadruple theory builds on many of the strengths of previous theories of love and it applies a temporal approach that has been proposed as the best way to understand love (Berscheid, 2010). It goes further than previous theories for several reasons. Firstly, it could potentially be applied to any form of love although, only brand, romantic and parental love were discussed in this paper due to the paucity of scholarly articles on other forms of love. One of the reasons current love scales and approaches have been unable to be applied in all forms of love (Hendrick and Hendrick, 1989; Whitley, 1993; Sternberg, 1997; Masuda, 2003; Graham and Christiansen, 2009), is because they capture only a part of the ACC model, unlike the quadruple framework which fully captures it.

Unlike previous theories, the quadruple theory’s application of the complex factor of connection/resonance gives it an edge in furthering our understanding of love. Proximity, positive shared experience, familiarity, and similarity are vital to connection and connection has the most profound influence on all the other factors.

Also, the dynamism and variation of these factors provide a fresh way to understand love from its development to collapse. As Figure 1 shows, love tends to take time to mature in a relationship and can die as these factors rise and decline. Figure 1 shows that variations in the presence of these factors represent different levels of love. Love in any relationship is influenced by the events in the environment it is embedded, and it responds favorably or negatively to these changes. Indeed, people get sick, old, lose their finances, travel in search of greener pastures creating distance, develop new interests different from their partner’s and all these influences the presence and absence of love. One brand becomes more innovative, improves its product quality and users experience over another and people gradually love it more than the one they previously loved. In other words, love is very dynamic and may be divided into high, moderate and low. Another point highlighted in Figure 1 is that the absence of one factor represents the absence of love and only the presence of all factors represents the presence of love. Indeed, the decline of a factor can be replenished in response to changes in the environment causing the reestablishment of love. Trust could decline but attraction and respect remain and over time trust could be replenished.

This dynamic understanding of love implies that it can be nurtured and sustained. As an example, for a brand to be loved and to maintain that love, it must make products that are attractive (appealing). It must be able to connect to its target customers by reaching out through adverts to achieve familiarity and it must ensure that its values, goals, actions are consistently similar to those of its customer base. Also, it must ensure its services and products and actions promote and maintain trust with its customers. It must respect (value) its customer’s interests and ensure that its services and products continue to receive the admiration of its customers. Table 2 describes how brand love can be nurtured and preserved.

**TABLE 2:** Brand love can be nurtured and maintained.

Brand love	Actions to nurture and maintain it
Connection	(1) Ensure that the values, goals, interests, etc. of the brand are similar or congruent to those of its customer base.
	(2) Ensure that customers are aware of its products and familiar with all new developments.
	(3) Ensure that customers use the brand as frequently as possible.
Attraction	Brand or product quality, value, aesthetic, innovativeness, etc. must be prioritized.
Respect	(1) Treats customers with the highest regard. (2) Ensure that its conduct and services take into consideration the concerns and interests of its customer base and address them. (3) Ensure that its products and services remain innovative and admirable.
Trust	Ensure that brand products and services, as well as conduct or actions, promotes and strengthens customers’ faith and confidence in the brand.

Using this framework, a love scale or algorithm could be developed to ascertain the presence or absence of love in any relationship. Such a scale must effectively capture these four factors and must consider the type of love being calculated in its approach. As an example, in trying to create a scale for romantic love, sexual attraction, and activity may be important for attraction and connection (depending on the age of the partners) but would be unnecessary in the calculation of brand or parental love.

## Major Challenges for the Theory

One of the biggest challenges the theory faces is the lack of psychometric data to prove many of its claims. Most of its arguments are based on decades of psychological data, but its lack of psychometric data weakens the theory significantly. Also, the entire premise of the theory is based on the ACC model, which has not been validated as essential or foundational to understanding love. Perhaps, something else needs to be added to the model that the theory may have missed. The argument that the quadruple theory captures the ACC model better than previous theories on love is an argument that has not been validated, and it remains to be seen if this is true. Also, the argument that it can be applied to all forms of love apart from the three discussed remains to be tested and verified.

## Conclusion

Gaps currently exist in our understanding of love and evidences from the existing literature show that a framework that can be applied to all forms of love is needed. The quadruple theory hopes to be that framework. It is likely to broaden our understanding of the complex nature of love. It could make love less complex by making it something that can be cultivated or nurtured, regulated and preserved. Future research should consider the modulatory roles of peptides, neurotransmitters, and hormones on these factors and their influence on love as well as the integrated parts of the brain that modulates all these factors and how they work synergistically in different stages of love.

It is important to note that love is universal and applies to people of all cultures, races, ethnicities, religion and sexual orientations. Indeed, romantic love as described by the quadruple theory applies equally to heterosexual relationships and to the relationships of people in the LGTBQ community.

In conclusion, culture has a monumental influence on what people feel, think, and how they behave toward other people and things in their environment (Karandashev, 2015; Ching Hei and David, 2018). So, it can be considered a modulating factor on the factors discussed and on love.

## Author Contributions

The author confirms being the sole contributor of this work and has approved it for publication.

## Conflict of Interest

The author declares that the research was conducted in the absence of any commercial or financial relationships that could be construed as a potential conflict of interest.
